# Supplementary figures and images for: Characteristics of new HIV diagnoses over 1995–2019: A clinic-based study in Montréal, Canada
Source: PLoS One. 2021 Oct 7;16(10):e0258383. doi: 10.1371/journal.pone.0258383 (PMC8496787; doi:10.1371/journal.pone.0258383)

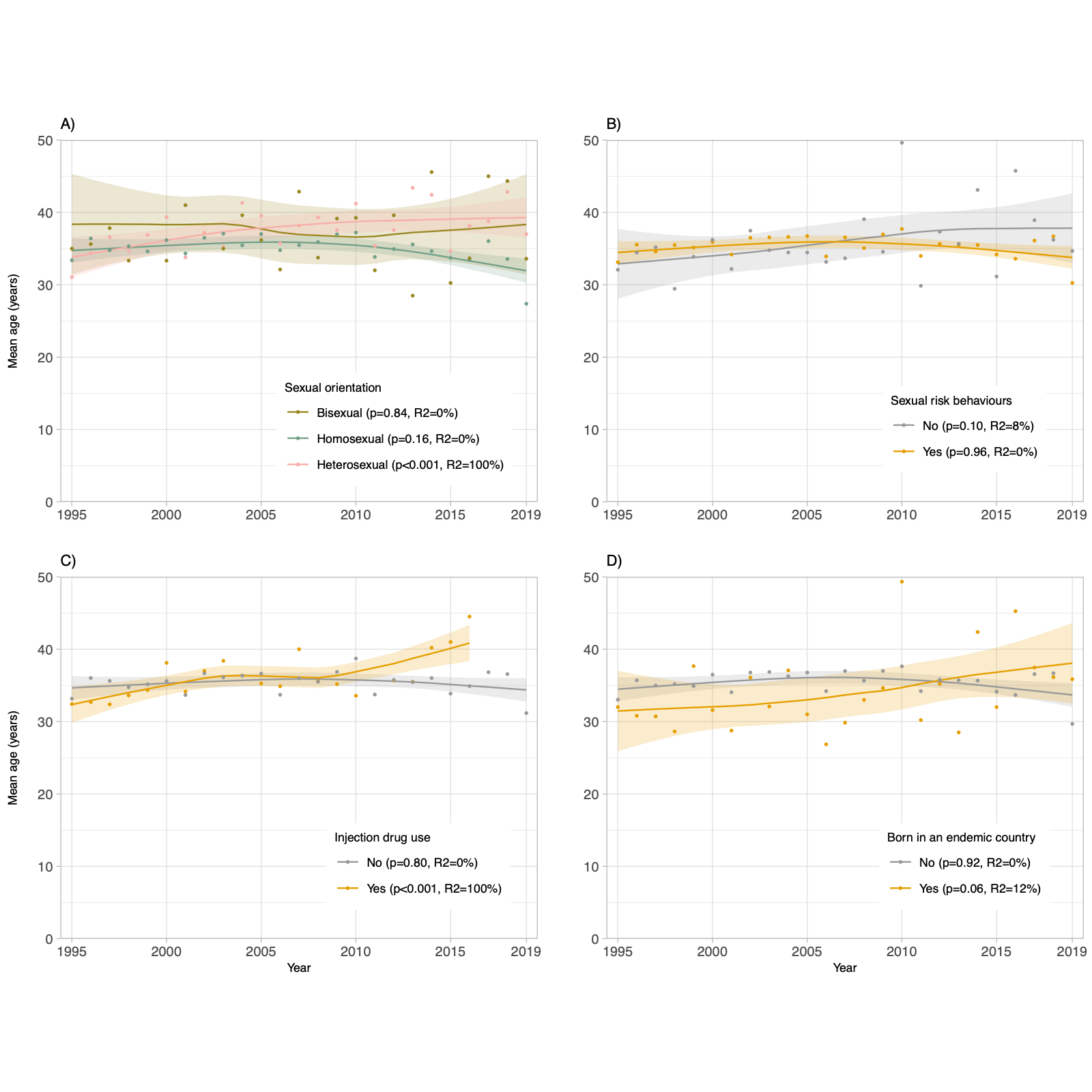

Supplement: S1 Fig — Panel A presents trends by sexual orientation group among men only (n = 2,342). Panels B to D present trends among combined men and women by sexual risk behaviours (including condomless sex, having a partner at risk, having an HIV-positive partner, having multiple sexual partners and/or having ever engaged in sex work), injection drug use, and origin (being born in an HIV-endemic country or not), respectively. Trends are displayed using local linear fitting and a degree of smoothing of 0.75. Time trends (p-values) were assessed by the mean of t-tests within univariate meta-regressions where the year of diagnosis was input as a continuous predictor variable. (TIF) [file pone.0258383.s001.tif]

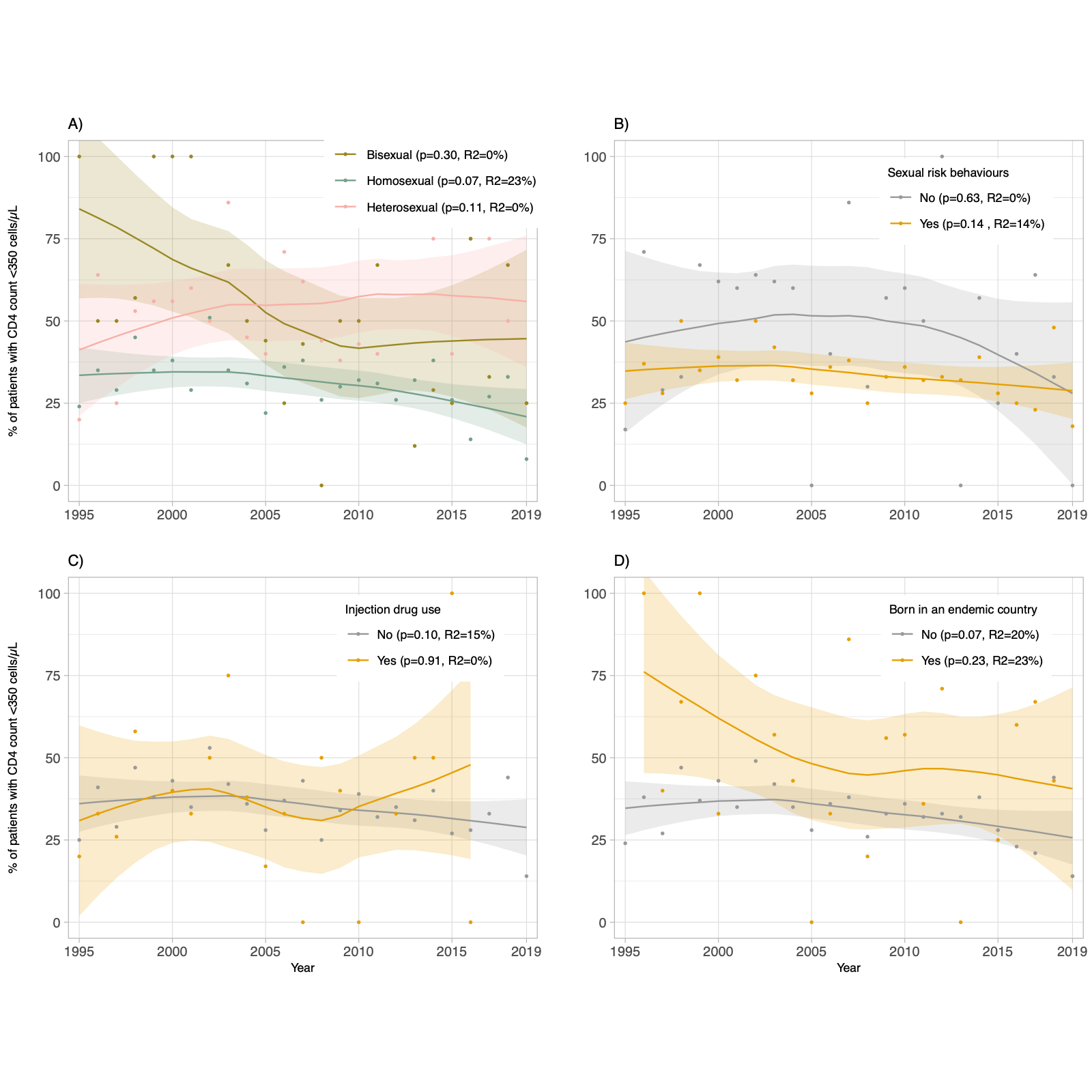

Supplement: S2 Fig — Panel A presents trends by sexual orientation group among men only (n = 1,737). Panels B to D present trends among combined men and women by sexual risk behaviours (including condomless sex, having a partner at risk, having an HIV-positive partner, having multiple sexual partners and/or having ever engaged in sex work), injection drug use, and origin (being born in an HIV-endemic country or not), respectively. Trends are displayed using local linear fitting and a degree of smoothing of 0.75. Time trends (p-values) were assessed by the mean of t-tests within univariate metaregressions where the year of diagnosis was input as a continuous predictor variable. (TIF) [file pone.0258383.s002.tif]

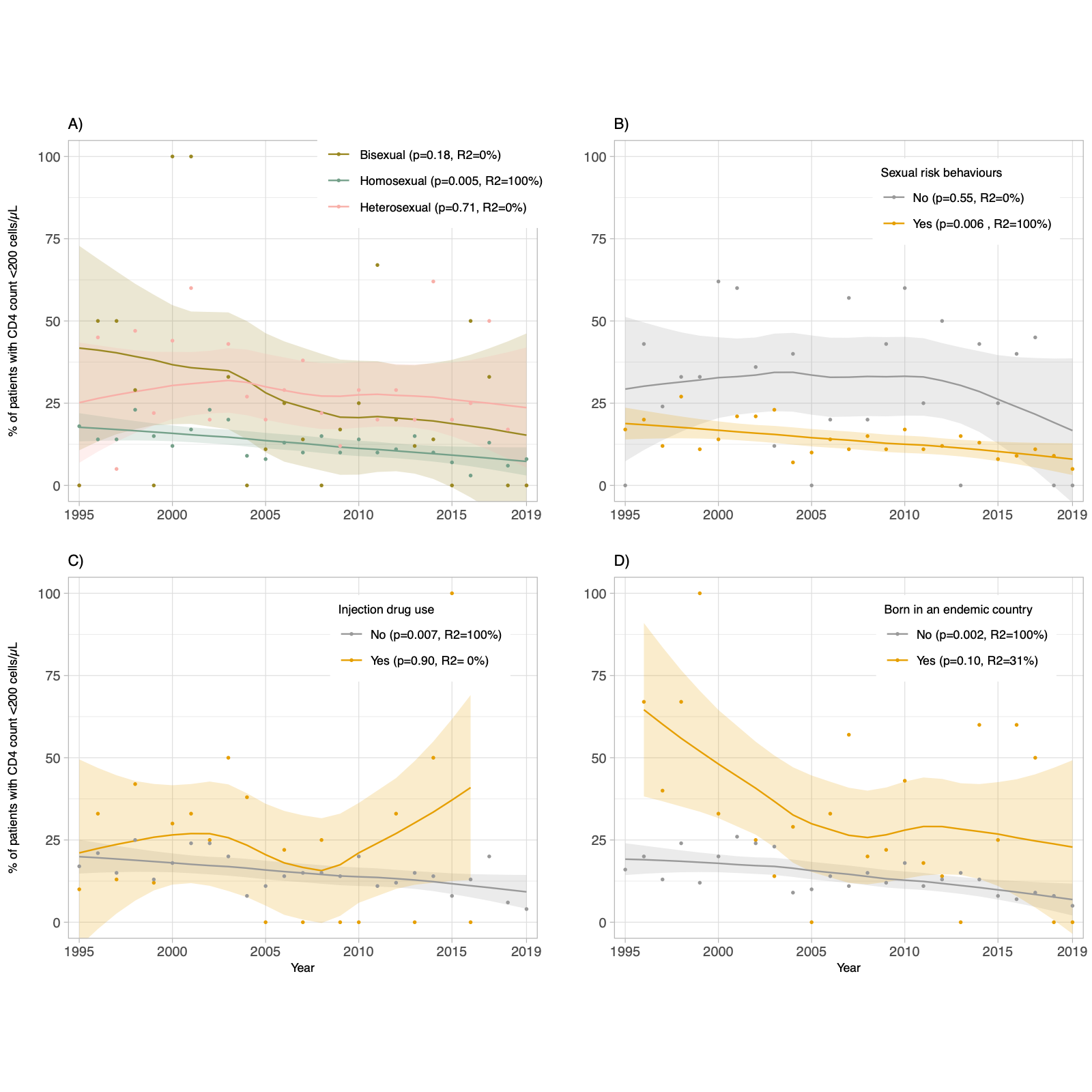

Supplement: S3 Fig — Panel A presents trends by sexual orientation group among men only (n = 1,737). Panels B to D present trends among combined men and women by sexual risk behaviours (including condomless sex, having a partner at risk, having an HIV-positive partner, having multiple sexual partners and/or having ever engaged in sex work), injection drug use, and origin (being born in an HIV-endemic country or not), respectively. Trends are displayed using local linear fitting and a degree of smoothing of 0.75. Time trends (p-values) were assessed by the mean of t-tests within univariate metaregressions where the year of diagnosis was input as a continuous predictor variable. (TIF) [file pone.0258383.s003.tif]
