# Supplementary material for: Characteristics of new HIV diagnoses over 1995–2019: A clinic-based study in Montréal, Canada
Source: PLoS One. 2021 Oct 7;16(10):e0258383. doi: 10.1371/journal.pone.0258383 (PMC8496787; doi:10.1371/journal.pone.0258383)
Supplement: S2 Table — (PDF) [file pone.0258383.s005.pdf]

S2 Table. Characteristics of patients at HIV diagnosis among participants to the HIV-positive cohort at the *Clinique médicale l'Actuel* in Montréal (Canada), stratified by availability of CD4 count and viral load at diagnosis (n=2,612), 1995-2019.

	Overall n = 2,612		With CD4 count n = 1,890		Without CD4 count n = 722	
	n	(%)	n	(%)	n	(%)
Mean age, years (SD)	35	(10)	36	(10)	34	(9)
Gender						
Male	2,342	(90)	1,737	(92)	605	(84)
Female [a]	270	(10)	153	(8)	117	(16)
Origin						
Caucasian	960	(37)	685	(36)	275	(38)
Black	244	(9)	149	(8)	95	(13)
Hispanic	183	(7)	123	(7)	60	(8)
Asian	66	(3)	49	(3)	17	(2)
Native	9	(0.3)	4	(0.2)	5	(0.7)
Other	51	(2)	37	(2)	14	(2)
Unknown	1,099	(42)	843	(45)	256	(35)
Sexual orientation						
Homosexual	1,876	(72)	1,431	(76)	445	(62)
Heterosexual	590	(23)	348	(18)	242	(34)
Bisexual	146	(6)	111	(6)	35	(5)
Exposure category [b]						
Sexual behaviours	2,293	(88)	1,717	(91)	576	(80)
Condomless sex	1,449	(56)	1,068	(57)	381	(53)
Having a partner at risk of HIV [c]	784	(30)	604	(32)	180	(2725
Having an HIV-positive partner	506	(19)	389	(21)	117	(16)
Having multiple sexual partners (>1)	680	(26)	552	(29)	128	(18)
Having ever engaged in sex work	73	(3)	54	(3)	19	(3)
Injection drug use	271	(10)	141	(8)	130	(18)
Born in an HIV-endemic country	219	(8)	132	(7)	87	(12)
Contaminated blood transfusion	6	(0.2)	4	(0.2)	2	(0.3)
Other [d]	10	(0.4)	5	(0.3)	5	(0.7)

SD: standard deviation

[a] Female participants include one transwoman who reported being homosexual and to have sex with men.

[b] Categories are not mutually exclusive.

[c] Partners at risk of HIV include people who inject drugs, HIV-positive individuals, individuals born in an HIV-endemic country, bisexuals, sex workers, and hemophiliacs.

[d] Other includes accidental exposure and mother-to-child transmission.
